# Transcriptional Regulation of Mononuclear Phagocyte Development

**DOI:** 10.3389/fimmu.2015.00533

**Published:** 2015-10-19

**Authors:** Roxane Tussiwand, Emmanuel L. Gautier

**Affiliations:** ^1^Department of Biomedicine, University of Basel, Basel, Switzerland; ^2^INSERM UMR_S 1166, Sorbonne Universités, UPMC Univ Paris 06, Pitié-Salpêtrière Hospital, Paris, France

**Keywords:** transcription factors, development, dendritic cells, macrophages, immunity

## Abstract

Mononuclear phagocytes (MP) are a quite unique subset of hematopoietic cells, which comprise dendritic cells (DC), monocytes as well as monocyte-derived and tissue-resident macrophages. These cells are extremely diverse with regard to their origin, their phenotype as well as their function. Developmentally, DC and monocytes are constantly replenished from a bone marrow hematopoietic progenitor. The ontogeny of macrophages is more complex and is temporally linked and specified by the organ where they reside, occurring early during embryonic or perinatal life. The functional heterogeneity of MPs is certainly a consequence of the tissue of residence and also reflects the diverse ontogeny of the subsets. In this review, we will highlight the developmental pathways of murine MP, with a particular emphasis on the transcriptional factors that regulate their development and function. Finally, we will discuss and point out open questions in the field.

## Introduction

The mononuclear-phagocyte system (MPS), which comprises dendritic cells (DCs), macrophages, and monocytes, is a heterogeneous group of myeloid cells. The complexity of the MPS is equally reflected by the plasticity in function and phenotype that characterizes each subset depending on their location and activation state. Specialized subsets of mononuclear phagocytes (MP) reside in defined anatomical locations, are critical for the homeostatic maintenance of tissues, and provide the link between innate and adaptive immune responses during infections. The ability of MP to maintain or to induce the correct tolerogenic or inflammatory milieu also resides in their complex subset specialization. Such subset heterogeneity is obtained through lineage diversification and specification, which is controlled by defined transcriptional networks and programs. Understanding the MP biology means to define their transcriptional signature, which is required during lineage commitment, and which characterizes each subset’s features. This review will focus on the transcriptional regulation of the MPS; in particular, what determines lineage commitment and functional identity; we will emphasize recent advances in the field of single-cell analysis and highlight unresolved questions in the field.

## The Mononuclear-Phagocyte System Network

As summarized in Table 1, the MPS is a rather heterogeneous group of myeloid cells, which includes DC, monocytes, and macrophages (1). DCs are mostly short lived and characterized by a half-life that varies between few days up to few weeks (2). This subset of MPs is equipped with pattern recognition receptors (PRR) and is specialized in antigen capture and presentation to T cells (3). At least three different DC subsets have been identified: plasmacytoid DCs (pDCs), and two common or conventional DC (cDC) subsets; cDC1, which express CD24, and CD8α in lymphoid tissues, or CD103 in peripheral organs; and cDC2, which express CD4, CD11b, and CD172 (1). This latter subset of cDCs is heterogeneous and seems to comprise also monocyte-derived DCs and activated macrophages, which have acquired a DC phenotype and most likely function (4).

**Table 1:** **Summarized are the three major murine MPs: dendritic cells, monocytes, and macrophages**.

MPs	Subset	Surface MK	Functions
Dendritic cells	pDCs	SiglecH, Bst2	Production of type 1 IFN (antiviral response)
	cDC1	XCR1, CD103/CD8, Clec9a	Th1 and CTL immunity, cross-presentation, IL-12 production
	cDC2	CD11b, Sirp-α	Th2 and Th17 immunity, production of IL-23 and IL-6
Monocytes	Ly6C high inflammatory	Ly6C hi CCR2 hi	Differentiate into DCs and tissue macrophages during inflammation
	Ly6C low patrolling	Ly6C low CCR2 low Cx3Cr1	Endothelial integrity
Macrophages	Tissue specific	F4/80, MerTK, CD64 CD11b	Tissue specific

*Each MP subset can be further subdivided into different subsets based on surface marker expression and function and as indicated*.

Steady-state monocytes are short-lived MPs. They are subdivided into two major subsets: patrolling and inflammatory monocytes, which are characterized by low and high expression of Ly-6C, respectively (5). Inflammatory monocytes are recruited and extravasate into infected tissues. They play a role in maintaining the correct inflammatory milieu, are important in the resolution of inflammation and in certain tissues monocytes will replenish the pool of resident macrophages (5–7). The role of patrolling monocytes is less clear but they are certainly involved in the homeostasis of the endothelium (8, 9).

The last subset of MP comprises the mostly long-lived tissue-resident macrophages (10). This subset is present in every developing as well as mature tissue, which is highly heterogeneous in terms of phenotype and function, reflecting the physiological needs of the organ of origin (11). Macrophages are thought to be required for the correct development and maintenance of tissues. This topological-related feature is possibly the reason for their extreme heterogeneity and their tissue specialization (12).

Collectively, MPs are highly plastic myeloid cells, which can perform very diverse functions. Table 1 summarizes the mostly used surface markers in mice and the function attributed to the different MP subsets.

## Transcriptional Regulation of Dendritic Cells Development

**Figure 1:**
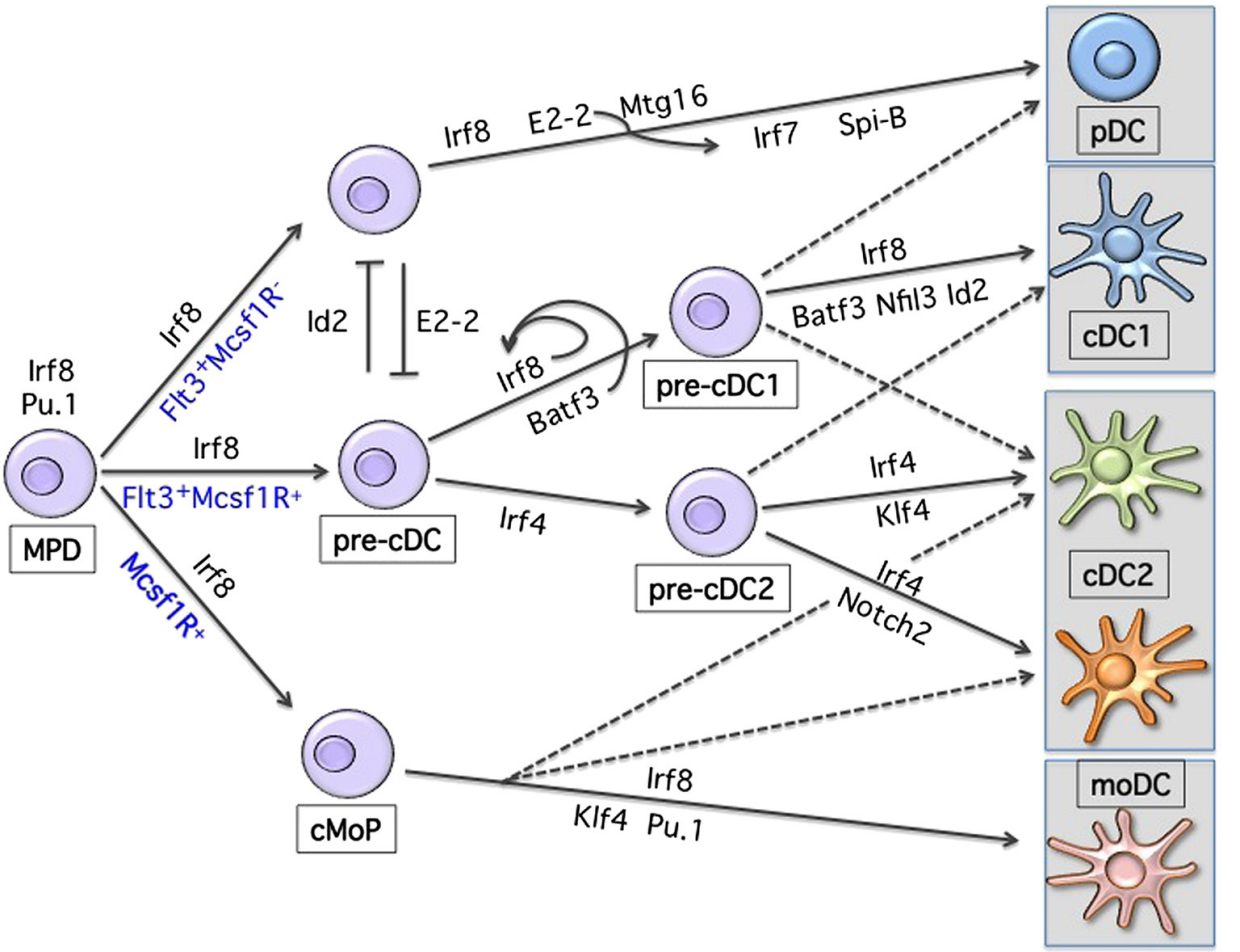
**Transcriptional development of dendritic cells**. Shown are the major transcription factors known to be involved in DC lineage commitment. Development occurs from a Flt3-, Irf8-expressing hematopoietic progenitor. Progressive acquisition of one or more TFs will result in differentiation toward a specific MP subset. Loss or reduction of one or more TFs can, to some extent, redirect commitment to another lineage.

As shown in Figure 1, lineage development of hematopoietic progenitor cells along DC lineage occurs through an orchestrated expression pattern of transcription factors (TF), yet the precise molecular mechanisms of lineage restriction and determination remains largely unexplained (2, 13–17). The analysis of gene-targeted mice has revealed the functional importance of a few critical TFs in DC development, with some of them affecting all DCs and some affecting specific subsets (18). DC progenitors are present within the fms-related tyrosine kinase 3 (Flt3)-expressing bone marrow fraction and sustained Flt3 signaling can be considered as instructive for DC development (19–22). Consistently, Flt3-ligand (Flt3L) supports the *in vitro* differentiation of progenitor cells into both pDCs and cDCs (23, 24). Genetic deletion of Flt3L, its receptor, or treatment of mice with Flt3 inhibitors leads to a 10-fold reduction of lymphoid-organ pDCs and cDCs (25, 26). Moreover, Flt3L injection or overexpression of Flt3L results in the expansion of both pDCs and cDCs in all lymphoid and non-lymphoid organs (27, 28). Engagement of Flt3 by Flt3L induces Stat3 phosphorylation and activation, identifying Stat3 as the critical checkpoint of Flt3-induced DC development and proliferation (29, 30). Mirroring Flt3 deficiency, Stat3-deficient mice have severely reduced DC progenitors and mature cells (29). Similarly, deletion of the transcriptional repressor growth factor independent 1 (Gfi1) results in impaired DC development (31). Gfi1-deficient mice show reduced Stat3 phosphorylation and nuclear translocation, with increased expression levels of the Stat3 negative regulators SOCS3 and PIAS3 suggesting that Gfi1 is downstream of Stat3 signaling in the Flt3-Flt3L-induced DC developmental pathway (31). However, the role of Gfi1 is more complex since mice deficient for this repressor show multiple hematopoietic impairments (32, 33). The defects related to Gfi1 deficiency can partially be related to dysregulation of Id2 expression (34–36). However, further studies using subset-specific deletion models will be instrumental to precisely dissect specific transcriptional requirements within the MP lineage. Similarly, despite the experimental evidence of DC expansion following sustained Flt3 signaling, the instructive mechanism promoting DC development is still unclear, given the broad expression of Flt3 on all short-term uncommitted hematopoietic progenitors (ST-HSC) (37, 38). A long non-coding RNA (lncRNA), named lnc-DC, was recently suggested to be the missing key element regulating Stat3 activity exclusively in DCs (39). lnc-DC RNA is expressed by mature DCs and by monocyte-derived DCs and seems to directly interact with Stat3 preventing its de-phosphorylation by SHP1. Furthermore, knockdown experiments of lnc-DC *in vitro* showed impaired DC development from mouse BM progenitors. The conservation of this lnc-DC in terms of function and of its consensus elements at the promoter region across species supports the hypothesis of a new level of regulation present in DC development. However, in mice the transcript seems translated into a highly expressed protein in adipose tissue (40). Further studies are therefore needed to understand potential species-specificities as well as its requirement *in vivo* under steady-state conditions.

**Figure 2:**
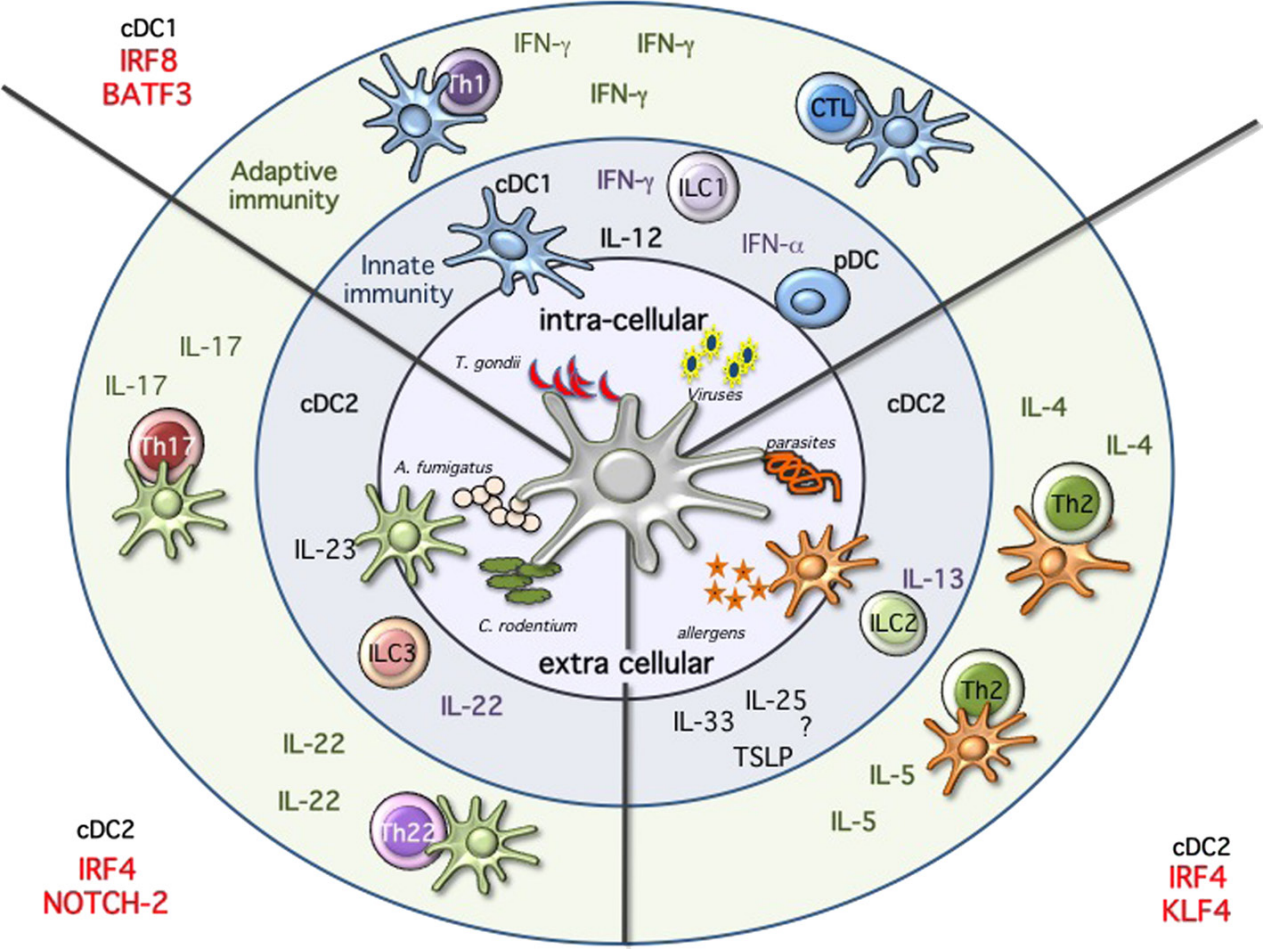
**Immune modules dendritic cells will sense the environment and start the immune response by producing cytokines, activating innate immune cells, and priming T cells**. Intracellular pathogens, such as *Toxoplasma gondii*, or viral infections will activate cDC1 and pDCs. These subsets are specialized in the production of IL-12 and type 1 IFNs, respectively. High amounts of IL-12 will activate innate lymphoid cells 1 (ILC1) to produce IFN-g and ultimately leading to the priming of Th1 immunity and sustain IFN-g secretion. Following viral infections, pDCs will produce high amounts of type 1 IFNs, while cDC1 will prime CTL response through cross-presentation of infected cells. Immunity against fungi and extracellular pathogens is mostly mediated by Irf4/Notch2 cDC2, which produce high amounts of IL-23, leading to the activation of ILC3 and IL-22 production as well as priming of Th17 and Th22 T cells. Th2 immunity during allergic reactions and following parasitic infections requires KLf4-dependent cDC2. In this case, the mechanism seems to be more complex and may require ILC2, but ultimately results in the activation of Th2 cells and the production of IL-4 and IL-5.

Proceeding along the DC developmental pathway, three major branches of mature DCs are identified: pDCs, CD24^+^ cDC1, and CD11b^+^ cDC2 (3, 16). pDCs and cDC1 both express and depend on the transcription factor interferon regulatory factor 8 (Irf8), while cDC2 express and are partially dependent on Irf4 (1, 18, 41–44). Despite major advances in our understanding of the transcriptional requirement during DC development, we are still unable to draw a clear developmental map (Figure 2) (13, 18). This may reflect subset heterogeneity as well as the plasticity, which characterizes DCs. Also, the expression of the different TFs is not unique and can change during differentiation and activation further complicating the picture.

During early stages of DC development, a progenitor that expresses high levels of Irf8 and shows developmental potential toward all DCs can be identified (42). It is likely that the first branching choice will determine whether pDCs or cDCs commitment occurs. The balance of the E-protein transcription factor 4 (Tcf4), also known as E2-2, and the E-protein inhibitor of DNA binding 2 (Id2) seems to determine lineage development toward pDCs or cDCs, respectively (45–50). Constitutive or inducible deletion of E2-2 in CD11c-expressing cells blocks the development of pDCs but not cDCs, while overexpression of Id2 inhibits pDC development (47, 48). E2-2 is required not only during development but also for lineage maintenance of pDCs (47, 51). Several targets of E2-2 have been identified such as SpiB, Irf8, and Irf7, and all contribute to pDCs lineage specification (47, 51). Despite the requirement for E2-2 during pDCs commitment, how Id2 and E2-2 are conversely induced and regulated is still an open question. Recently, the eight-twenty-one (ETO) protein 2 or Mtg16 (also referred as core-binding factor, runt domain, alpha subunit 2, translocated to 3 Cbfa2t3) was suggested to target and repress Id2 together with E2-2 and inhibit Irf8-expressing cDC1 development, while favoring pDC commitment (52). Consistently, Id2 and Mtg16 double-deficient mice show restored pDC potential (52). However, Mtg16 seems to act together with E2-2 leaving the question on how lineage determination toward E2-2- or Id2-expressing progenitors occurs, still open. On the other side, one other candidate, which could be involved in reinforcing lineage fate toward Irf8-expressing cDC1 at the expenses of pDCs could be the leucine zipper transcription factor E4BP4, also referred as Nfil3 (53). Mice deficient for this TF show increased pDC and reduced cDC1 development (53). The mechanism of action remains to be elucidated since Id2 expression does not appear to be perturbed and only the basic leucine zipper transcription factor ATF-like 3 (Batf3) expression was shown to be reduced (53). Phenotypically, a bias toward pDC development has been observed within the macrophage-colony-stimulating factor receptor (M-CSFR) negative progenitors, whereas cDCs precursors are enriched within the M-CSFR expressing BM fraction (54, 55). These results may suggest that under sustained M-CSF stimulation uncommitted progenitors may lose the potential toward pDCs. Alternatively, as recently suggested, the absence of GM-CSF signaling, which induces STAT5 phosphorylation, could be the permissive condition to promote pDC development (56). Accumulation and/or withdrawal of specific cytokines during proliferation and differentiation as well as regulation of TF levels through division of progenitor cells could partially explain how BM niches influence development and lineage commitment (57, 58).

Proceeding along DC development, a common cDC progenitor able to differentiate *in vivo* into both CD24^+^ cDC1 and CD11b^+^ cDC2 was identified (55, 59–61). And recently, lineage-tracing studies allowed further dissection of cDC commitment and resulted fundamental to establish the transcriptional requirements during development of clonogenic cDC progenitors (62, 63). The expression pattern of the zinc finger and BTB domain containing 46 transcription factor Zbtb46 (also called Btbd4) can be considered as cDC-lineage specific within hematopoietic cells (64–66). This TF is not present on pDCs and is induced on monocyte-derived DCs, supporting on the one hand early divergence of pDCs during DC commitment, and on the other hand suggesting a developmental convergence between cDCs and monocyte-derived DCs (4, 66). Similarly, lineage-tracing experiments were performed using mice expressing Cre recombinase under the control of Clec9a also referred as Dngr-1 (67). Although labeling is not absolute on all cDCs subsets, it seems to be restricted to pre-cDC progeny, without marking inflammatory-derived DCs (67). The use of these reporter mouse models will help us better characterize the ontogeny of specific cDCs subsets also depending on the tissue of origin and whether under steady-state or inflammatory conditions.

The CD24^+^ cDC1 branch of cDCs depends on the transcription factors Irf8, Id2, Nfil3, and Batf3 (68). The generation of mice deficient for Batf3 has revealed the common origin and the lineage identity of Irf8-expressing cDC1 cells, also referred as CD8a^+^ or CD103^+^ across all lymphoid and peripheral organs (69, 70). However, while only Irf8 was shown to be necessary for commitment, Id2, Nfil3, and Batf3 are dispensable under certain conditions (71, 72). Lineage choice seems influenced by high and sustained levels of Irf8 during cDC1 commitment. Binding of Batf3 and Irf8 to an AP1-IRF composite element (AICE) within the Irf8 super-enhancer in CD24- or Zbtb46-gfp-expressing immediate progenitors leads to sustained Irf8 expression and cDC1 development (62). In the absence of Batf3, reduced Irf8 levels, redirect commitment of a CD24-expressing cDC1 progenitor toward the Irf4-expressing cDC2 lineage (62).

Despite the recent advances, how the branching of cDC1 and cDC2 occurs is still an open question. The recent identification of a committed cDC2 progenitor might help to identify the key factors involved in this process: we still need to understand how expression of Irf4 progressively replaces Irf8, and how those two TF determine the identity of these subsets. Furthermore, the cDC2 lineage, as already mentioned, is highly heterogeneous and possibly contains multiple subsets (1, 2, 11, 16, 18). Mature CD11b-expressing cDC2 express high levels of Irf4, suggesting an important role for this TF within this lineage. And indeed, absence of Irf4 impairs the development as well as the function of cDC2 (42, 44, 73–77). In mice lacking IRF4 in CD11c-expressing cells, cDC2 numbers are reduced in lung and small intestinal DCs, while no difference is reported for skin (44, 74). However, reduction in lung and lamina propria cDCs is only observed upon deletion of Irf4 in early progenitors (44; 74). Despite, normal numbers of skin DCs in Irf4-deficient animals, migration to draining lymph nodes is impaired as a consequence of defective induction of CCR7 (78). Furthermore, reduced up-regulation of MHC-II and co-stimulatory molecules is also associated with Irf4 deficiency (75, 77, 78). Collectively, Irf4 shows a broad action across different tissues and potentially subsets, and further studies are required to be able to understand the specific requirement of this TF during development.

Other TFs reported to display a reduction of cDC2 are RelB, Notch2, RbpJ, and the Kruppel-like Factor 4 (Klf4) (79–85). Notch2 is required for terminal differentiation of endothelial cell-selective adhesion molecule (ESAM)-expressing splenic cDC (81, 83). Similar to Notch2 deficiency, mice compromised in Runx3 (86, 87) and in the alternative NF-kB pathway show a reduction in the development of ESAM^+^ cDCs (80, 88). However, a survival disadvantage in competitive settings appears to be present in mice with compromised NF-kB signaling, suggesting caution in proposing the requirement for NF-kB during DCs development (80, 88). Klf4 deficiency results in impaired development of the so-called “double negative” DCs in skin draining LN and a partial reduction of Sirp-α but not splenic ESAM-expressing cDC2 across all the organs (84). In these mice, cDC progenitors are impaired in their ability to down-regulate Irf8 and up-regulate Irf4. However, the *in vitro* differentiation potential of Irf4-expressing cDCs as well as expression of Irf4 on peripheral cDCs is not compromised. This can be explained by the existence of at least two cDC2 subsets, where only the Klf4/Irf4-dependent one is developmentally impaired. Alternatively, a different maturation/activation state, which requires Klf4, may exist within the Irf4-expressing cDC2 subset.

Collectively, a partial reduction associated with the lack of one or the other TF confirms the developmental, and supports the subset-specific heterogeneity observed in single-cell sequencing experiments for the Irf4 and CD11b-expressing cDC2 cells (89–92). The transcriptional diversity, which characterizes these cDC2 cells, results and reflects a functional heterogeneity (Figure 2). Notch2 cDC2 are required for anti bacterial Th17/IL-22 immunity, while Klf4 deficiency results in impaired Th2 immunity (83, 84, 93). Expression of Irf4 in cDCs is necessary for both Th17 and Th2 responses further highlighting the complexity of this TF in DC biology (44, 73, 74, 77). Understanding whether the absence of a subset or a functional defect caused by a transcriptional deficiency on the remaining subset could account for the observed phenotypes will require subset-specific deletion. Furthermore, we also need to explore more in detail the influence of tissues on the different subsets. Are tissue-specific cues driving the expression of a transcriptional signature in a similar way as recently revealed for macrophages? (12) Are the differences reflecting a developmental or a functional heterogeneity? Is a developmental convergence between cDCs and monocyte-derived DCs creating the confusion within this branch of cDCs. We need a better characterization of the different subsets, which fall under the broad umbrella of CD11b or Irf4-expressing cDC2 and some progress has certainly been made with the introduction of new reporter mice as previously discussed as well as the recently identified committed progenitor. Teasing this heterogeneous pool of Irf4-expressing cDC2 apart is currently an active field of investigation (90, 91, 94). And new technologies will be instrumental to improve our comprehension of the molecular clues, which regulate lineage commitment. A recent report analyzed stage and subset-specific expression of mi-RNAs during DC development and miR-142 was identified as a key regulator of cDC2 differentiation, further adding additional complexity to our current understanding of DC development (91).

Better genetic models are needed and will possibly be soon developed as a result of the recently published single-cell analysis (16, 89). Identifying TFs or surface markers, which would compromise or trace the development of one lineage independently of the anatomical localization, as previously done in Batf3^−/−^ mice for Irf8-dependent cDC1 would be of great advantage (69, 70).

## Transcriptional Regulation of Monocyte Development

The molecular regulation, which defines monocyte differentiation and lineage commitment, is poorly understood (95). Most of the identified TFs, that result in impaired monocyte development, also show an effect on other hematopoietic lineages. The transcription factors Irf8, Sfpi1 (PU.1), Egr-1, Stat3, Gfi1, Gata2, Gbx2, Nur77, retinoic acid receptors, C/EBPα and C/EBPβ, Klf4, and c-Maf as well as members of the NF-κB family members are all involved in monocyte differentiation, however their function is often redundant, certainly not limited to monocytes and in some cases mediating proliferative and/or survival rather than instructive cues (96). Most of the TFs involved in monocyte differentiation are shared within the myelo-monocytic branch. Some of them were already mentioned as important during DC development; others are involved in macrophage and or granulocyte commitment; we are therefore aware that we can only provide here a simplified transcriptional path, which leads to monocyte development and that more efforts are required to better understand.

Expression of the ETS family transcription factor Sfpi1 or PU.1 at early stages is suggested to antagonize on the one hand key regulators of other developmental pathways, such as GATA-1 for erythroid lineage, and on the other hand activate myeloid-specific factors such as Irf8, Klf4, and Erg1 (95). A critical step in monocyte differentiation is the induction of Csf1R expression at the cell surface. This seems to be regulated by Klf4 and Irf8, however both factors are also involved in cDC development, as previously discussed, (85). Furthermore, Csf1R is also needed for macrophage development.

The identification of a committed progenitor with monocyte-restricted potential called cMoP confirmed high expression levels of the above-mentioned TF (97). However, none of those is unique to monocyte differentiation and potentially complex genetic models will be required to unravel the transcriptional map required for monocyte lineage specification.

## Origin of Tissue-Resident Macrophages

**Figure 3:**
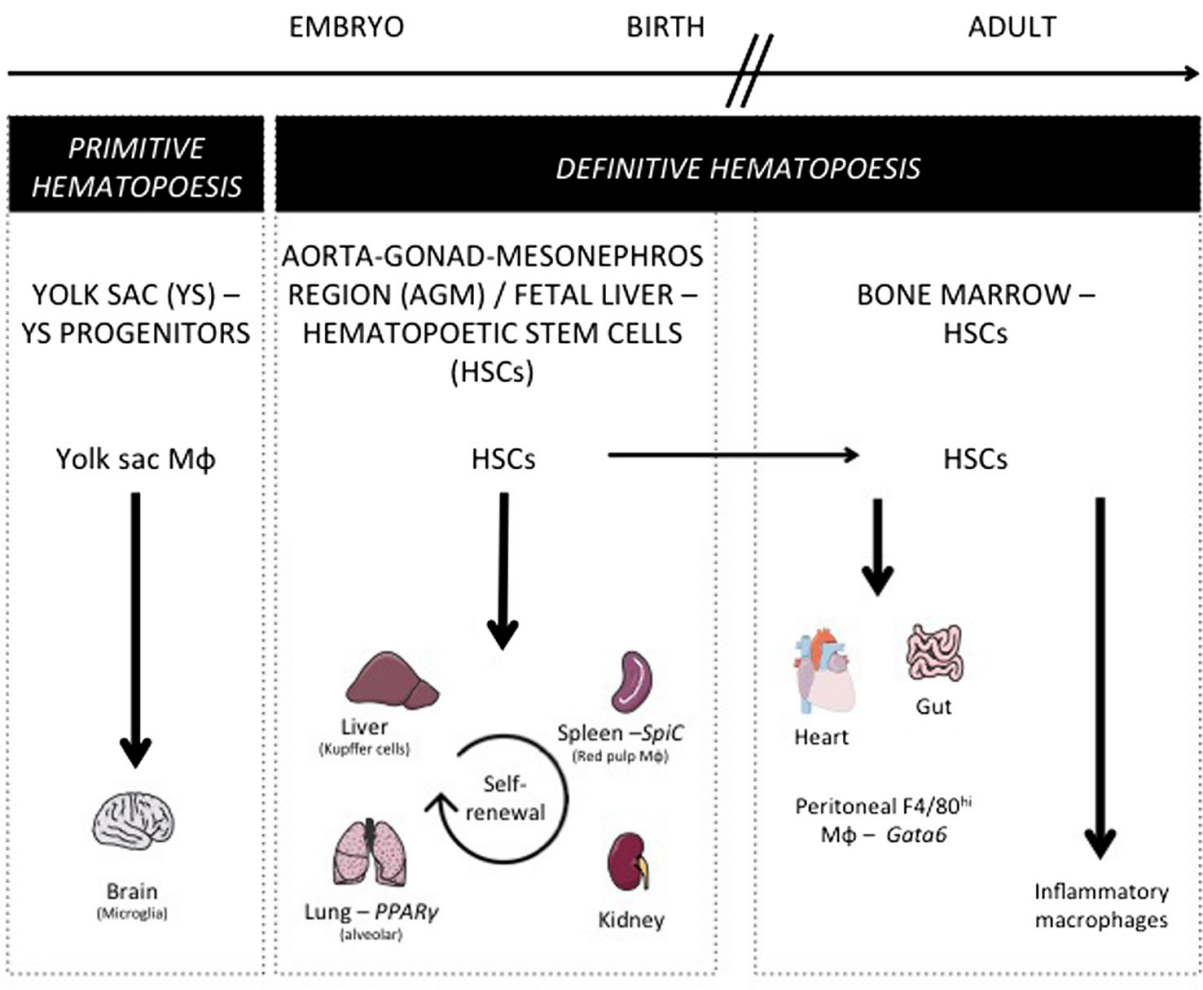
**Macrophage ontogeny and requirement on specific transcription factors**. This figure shows the progenitors of the different tissue-resident macrophage populations. During embryogenesis, yolk sac progenitors only give rise to microglia. Most tissue-resident macrophages develop before birth and are derived from early hematopoietic stem cells (HSC) present in the aorta–gonad–mesonephros region and in fetal liver. After birth and in the adult mouse, alveolar, splenic, liver, and kidney macrophages maintain their pool by self-renewal, while gut and heart macrophages need a constant replenishment from bone marrow HSC. Additionally, this figure depicts the transcription factors specifically controlling a single resident macrophage population such as Spi-C for splenic red pulp macrophages, PPARγ for alveolar macrophages, and Gata6 for peritoneal F4/80hi macrophages.

As discussed above for DCs, similar questions arise considering tissue-resident macrophage origin and development. Lineage-tracing studies recently revisited their origin and revealed how their maintenance in adult tissues is mostly independent from monocytes and adult definitive hematopoiesis (10). Indeed, tissue-resident macrophages were proposed to develop from a Myb-independent but Sfpi1 (PU.1)-dependent fetal progenitor present in the yolk sac (YS) (5, 6, 15, 98–100) and capable of seeding the developing embryo and self-renewing during adulthood. This developmental path was first described for microglia, the brain-resident macrophages (98, 99), but still remained elusive for a number of other macrophage populations. Using similar tools, the contribution of YS progenitors to a number of adult tissue-resident macrophage populations was next assessed and only very limited input was found in most tissues tested (101). In parallel, other studies conducted in the lung and skin found that resident alveolar macrophages and Langerhans cells originated from fetal monocytes (99, 102). A recently described hypothesis is now trying to bridge these findings by proposing the existence of erythro-myeloid progenitors (EMP) distinct from hematopoietic stem cells (HSCs), which develop in YS (E8.5) and colonize the fetal liver at E16.5 giving rise to fetal erythrocytes, macrophages, granulocytes, and monocyte (103, 104). Such progenitors would generate microglia early during fetal development and participate to Kupffer cells and Langerhans cells development, but its definitive participation to the generation of other tissue-resident populations, as well as its long-term persistence, still remains to be firmly established. Indeed, a very recent study is now arguing that fetal HSCs, and not YS progenitors or EMPs, give rise to most tissue macrophage populations, except microglia known to originate from YS progenitors other than HSCs (105). This study also highlighted that while most tissue macrophages subsets maintain by self-renewal in the adult, peritoneal, dermal, and colonic residents macrophages needed continuous HSCs input to be maintained during lifetime. Accordingly, gut macrophages, most likely a specific population of macrophage residing in the serosa (106), were shown to derive from HSC-derived circulating monocytes (107). Moreover, blood monocytes can participate to the maintenance of heart macrophages in the adult (108). During inflammation, in addition to tissue-resident macrophages, some macrophages found in tissue differentiate from locally recruited Ly-6C^hi^ monocyte. Such monocyte-derived macrophages reside only for a short period of time in the tissue until inflammation resolves, and are cleared through local cell death (109). Overall, these studies suggest that there is probably more than a single developmental pathway to generate tissue macrophages and to support their self-renewal potential and unique long-term maintenance ability (Figure 3).

## Pathways Allowing for Tissue-Resident Macrophage Development and Maintenance

Proceeding along development, Runt-related transcription factor 1 (RUNX1) is required at early stages of myeloid lineage specification and regulates the expression of Sfpi1 (PU.1) which has to be expressed at high levels to allow for development and maintenance of macrophage differentiation (57). One of the most crucial target genes of PU.1 during macrophage development is *Csf1r*, which encodes the receptor for M-CSF and IL-34 (110). Signaling of M-CSFR through either M-CSF or IL-34 allows for the maintenance of tissue-resident macrophages (111, 112). Other TFs required for macrophage development, which co-operate with PU.1 in lineage determination, are AML1 and CCAAT enhancer-binding proteins (C/EBP) (113, 114). Overall, our understanding of the molecular pathways controlling tissue macrophage development in general, as well as their maintenance, remains poorly defined and further studies are need to better characterize how their development is regulated. While for DCs, deletion of a subset might result in minor consequences, macrophages are thought to be critical for the organogenesis and organ homeostasis, therefore deletion of a subset could be deleterious for the life of the individual or only compatible with compensation through alternative subsets or pathways.

## Tissue-Resident Macrophages Diversity and Tissue-Specific Transcription Factors Controlling Resident Macrophage Development and Maintenance

Our analysis of the transcriptional landscape of tissue-resident macrophages revealed wide heterogeneity across tissues, leading to the definition of population-specific signatures. These specific signatures were recently shown to rely on distinct enhancer landscapes shaped by the tissue microenvironment (115, 116). Using the Immunological Genome database, the reconstruction of lineage-specific regulation from gene-expression profiles across lineages (117) revealed gene modules selectively associated with a single tissue macrophage population (12). Additionally, TFs were predicted to regulate these modules, and thus could potentially influence the development of resident macrophages in a tissue-specific manner (12). Among others, predicted regulators included Spi-C for red pulp macrophages, which confirmed precedent findings (118) and thus validated the predictive power of the algorithm. Indeed, Spi-C is a TF closely related to Sfpi1 and highly expressed in spleen red pulp macrophages compared to other phagocytes (12, 118). Mice deficient for Spi-C lack splenic red pulp macrophages (118), leading to defective red blood cells recycling and iron accumulation in the spleen. At which levels Spi-C acts to control the differentiation and/or survival of red pulp macrophage remains to be determined. LXRα is another TF needed for splenic red pulp macrophage development (119), and whether Spi-C and LXRα interact together in this process is not known. Interestingly, intracellular heme accumulation following erythrocytes uptake induced Spi-C expression by stimulating the degradation of its transcriptional inhibitor Bach1 (118). Thus, heme-induced Spi-C controls the functionality of splenic red pulp macrophages, but also their maintenance albeit by an undetermined mechanism.

Similarly, PPARγ was identified as a regulator for lung macrophages (120). It is a ligand-controlled TF of the nuclear receptor family known for its role in lipid metabolism (121). Previous work has shown that PPARγ expression is important to maintain lung macrophages functionality and surfactant catabolism (122). We reported that conditional deletion of PPARγ in lung macrophages strikingly altered their transcriptome (120). Dysregulated expression of a number of genes involved in lipid metabolism was observed (120), and many of these genes were known targets of the sterol-responsive transcription factor LXR. Accordingly, increased sterol accumulation was observed in lung macrophages lacking PPARγ, as well as decreased expression of genes involved in inflammation and immunity (120). Using a different gene deletion approach, it was recently shown that PPARγ could also be key in controlling the development of this subset. Such discrepancy between models might relate to the different temporal induction of the cre expressing strains used in these two studies (123).

Finally, GATA6 was identified as a specific peritoneal macrophage regulator and we observed that its expression was selectively found in F4/80+ peritoneal macrophages across many lineages tested (12), suggesting that it may represent the master regulator of tissue-resident peritoneal macrophages. Interestingly, GATA6 expression by resident peritoneal macrophages was dependent on retinoic acid signaling *in vivo* (12) and mice lacking GATA6 in macrophages, generated by crossing Gata6^fl/fl^ mice with Lyz2-cre, showed a strong reduction in F4/80+ peritoneal macrophages (115–117). Additionally, Th2 inflammation following parasitic infection failed to increase peritoneal macrophage numbers in Lyz2-cre × Gata6^fl/fl^ mice (124), as described for wild-type mice (125). Impaired steady-state numbers of peritoneal macrophages in the absence of GATA6 was accompanied by impaired self-renewal, marked increased in S/G2-M cell cycle phases and accumulation of multinucleated macrophages due to impaired cytokinesis (126). While reduced survival of peritoneal macrophage already explains the strong contraction in their number, impaired cytokinesis will likely further exacerbate the phenotype. GATA6 deficiency in F4/80+ peritoneal macrophages led to the down-regulation of Aspa mRNA, which encodes an aspartoacylase generating acetyl-CoA, a central cellular metabolite, from *N*-acetylaspartate (124). Interestingly, mice lacking Aspa showed reduced F4/80+ peritoneal macrophages. Overall, a tissue-specific transcriptional network driven by GATA6 controls multiple pathways all required for the maintenance of F4/80+ peritoneal macrophages.

## Concluding Remarks

In the past few years, major advances have been made in our understanding how the development of myeloid cells occurs. DNA, RNA protein sequencing and characterization on entire tissues and populations is now a more accessible technology. This combined with improved multicolor flow cytometry and CyTOF technology has allowed us to better understand which TFs identify specific subsets and developmental stages during hematopoietic development (89, 90, 124, 127). However, as it is often the case, the better our analysis tools become, the more complex the picture appears. And despite these advances, we are now starting to perceive how many more gaps need to be filled in order to be able to draw a definitive road map for every MP subset. Significant progress has been made in defining, which TF are needed during DC and macrophage development in specific tissues. Monocyte development, however, is still elusive and most of the factors identified rather compromise their survival, making hard to discriminate between developmental and survival defects. Moreover, in the past few years, it has become obvious how tissues are able to influence not only the phenotype but also the function of the different subsets. This observation translates in changes in the transcriptional signature, which identifies each subset in a given tissue. Tissue-associated hallmarks have been mostly studied in macrophages, however profound consequences appear to matter also within DC subsets. It will therefore be important to discriminate between tissue- versus subset-specific transcriptional identity, to define intrinsic properties, and functional potential for every subset across and within the different tissues. On the one hand, it is attractive to think that subset specialization similar as for T and innate lymphoid cells is also present within the myeloid compartment. On the other hand, we are aware that myeloid cells are characterized by an elevated intrinsic functional plasticity. Anatomical compartments, pathogen and antigen dose as well as small micro-environmental cues might drastically influence the phenotype, the transcriptional landscape as well as the function of the different subsets during immune responses and we are just starting to explore in depth the complexity of the different subset in response to an immunological insult (90, 127). For DCs, a model has been recently suggested which takes into account the development of a subset with its immunological function. As shown in Figure 2, expression of Irf8 and Batf3 is needed in response to pathogens or immunological conditions where IFN-γ is required. On the other side, Irf4 is essential to stimulate Th17, Th2, and IL-22 responses. Within the Irf4 response, Notch2 and Klf4 are specifically required for Th17/IL-22 or Th2 immunity, respectively. The scenario, which appears, is consistent with functional modules of transcription across different cell types, i.e., Klf4 is also necessary for goblet cell development and polarization of M2 macrophages, whereas Notch is required for ILC3 development. Similarly, Nfil3 is important not only for cDC1 but also for NK and ILC1 cells development.

Several TFs that are required for MP development have been characterized; we can draw a map for their temporal requirement along development but for most of them the precise mechanism of action and their targets still need to be identified. Furthermore, since the developmental as well as the functional requirements for a transcriptional pathway are most often shared, caution is necessary to ascribe a specific role to a TF. Recently, a Waddington landscape was suggested to explain the plasticity in DC development (92). A similar concept may reflect and be applied to the entire MP system, where lineage commitment, specific functions, as well as subset identity could depend on the achievement of a threshold of a pool of TFs, rather than a unique master regulator. This concept would explain the so-called “graded-commitment” obtained from barcoding individual progenitors and performing lineage-tracing experiments (128). A second level of complexity is characterized by the fact that multiple subsets share the same TF, though the functional requirements are different. For example, Irf4 seems to regulate migration in skin DCs but not in other peripheral tissues, such as lungs. The functional outcome might be shared; such as in both cases antigen presentation is impaired, however it is important to understand the different requirements depending on the tissue of origin.

The study of MP is characterized by blurry phenotypic boundaries, which do not allow for unequivocal identification of the different subsets. The absence of specific markers leads to the absence of specific genetic tools and sometimes conflicting or unclear results are present in the literature. For lineage-specific deletion within cDC, we still relay on CD11c–cre mice, despite the evident limitations of this model. For monocytes as well as macrophages we lack genetic models, which would allow for selective and specific depletion of inflammatory or patrolling monocytes as well as tissue macrophages. Efforts to generate better lineage-deleter mouse models are therefore required and should be a priority in the next future to better understand the development as well as the contribution of MPs during an immune response.

## Conflict of Interest Statement

The authors declare that the research was conducted in the absence of any commercial or financial relationships that could be construed as a potential conflict of interest. The Guest Associate Editor Martin Guilliams declares that, despite having published one manuscript together with Roxane Tussiwand, the review process was handled objectively.
